# B-cell Acute Lymphoblastic Leukemia in a Patient With Crohn’s Disease After Anti-TNF-Alpha Treatment

**DOI:** 10.7759/cureus.77400

**Published:** 2025-01-13

**Authors:** Abded Alraouf Kawtharani, Laura Akiki, Abbas Rachid, Antoine S Geagea, Antoine Abi Abboud

**Affiliations:** 1 Gastroenterology and Hepatology, Faculty of Medicine, Lebanese University, Beirut, LBN; 2 Internal Medicine, Faculty of Medicine, Lebanese University, Beirut, LBN; 3 Gastroenterology and Hepatology, Lebanese University, Lebanese Hospital Geitaoui University Medical Center, Beirut, LBN

**Keywords:** anti-tnf-α, b cell acute lymphocytic leukemia, crohn’s disease, ibd associated cancer, immunosuppressive therapies

## Abstract

This case report details a patient with Crohn's disease (CD) who developed acute lymphoblastic leukemia (ALL) 16 years after their initial diagnosis. Various immunosuppressive therapies used in the treatment included azathioprine, infliximab and, for a short period, vedolizumab and steroids for CD and hyper-CVAD chemotherapy for leukemia. The association between CD and increased cancer risk, particularly hematological malignancies, emphasizes the importance of regular cancer surveillance, including hematological assessments, for patients on immunosuppressive therapy.

## Introduction

Crohn’s disease (CD) is a chronic inflammatory bowel disorder characterized by relapsing and remitting inflammation of the gastrointestinal tract [1]. Its etiology remains complex and multifactorial, including genetic predisposition, environmental factors, and dysregulated immune responses [2]. The association between inflammatory bowel disease (IBD) and the onset of cancer arises from persistent inflammation and the extended use of immunosuppressive or biologic treatments, including thiopurines and anti-tumor necrosis (TNF)-alpha agents [3,4].

While abnormalities affecting all hematopoietic cell lines can manifest in individuals with IBD, anemia is considered the predominant hematologic complication. Nevertheless, individuals with IBD may have thrombocytosis and consequent thromboembolic events because of an enhanced coagulative state. Additionally, immunosuppressive regimens involving thiopurines, either independently or in conjunction with anti-TNF-alpha agents, can cause leukocytosis, thrombocytopenia and, in prolonged scenarios, the emergence of acute myeloid leukemia and severe myelodysplastic syndromes (MDS) as adverse outcomes [3,5].

In this case report, we present an uncommon incidence of coexisting CD and acute lymphoblastic leukemia (ALL), particularly Philadelphia chromosome-positive ALL (Ph (+) ALL), shedding light on the complex relation between IBD and hematologic malignancies. Additionally, we discuss potential ramifications for the management of both conditions, taking into consideration the influence of CD and its treatment approaches on the onset and progression of hematologic disorders. Appreciating this correlation is crucial for defining therapeutic approaches and offering care plans for individuals with diagnosis of both CD and hematologic malignancies.

## Case presentation

In January 2008, a 43-year-old male patient presented with recurrent abdominal pain, distension, diarrhea, and weight loss. Imaging via CT scan revealed dilated ileal loops and moderate thickening of the terminal ileum wall. Following an ileocolonoscopy and biopsy, CD was diagnosed, characterized by the presence of multiple aphthoid ulcers and partial ileocecal valve stenosis. The patient was classified as A2-L3-B2 according to the Montreal classification. Treatment was initiated with azathioprine and mesalamine; however, adherence to the prescribed regimen was challenging.

In 2012, exacerbation of symptoms led to further investigation, revealing erythematous mucosa in the gastric antrum and body upon gastroscopy, while colonoscopy uncovered rectal to cecal friability and superficial ulcers. Despite maintenance of the existing treatment regimen, efforts were made to enhance patient adherence. By 2014, severe symptoms recurred, characterized by the presence of multiple aphthoid ulcers extending from the rectum to the cecum, in addition to persistent ileocecal valve stenosis. Infliximab therapy was initiated, and it led to favorable symptom control until 2020, when recurrent symptoms prompted antibody testing, showing elevated levels. Subsequently, vedolizumab was initiated until its discontinuation due to its unavailability, necessitating the utilization of prednisone until February 2024.

**Figure 1:**
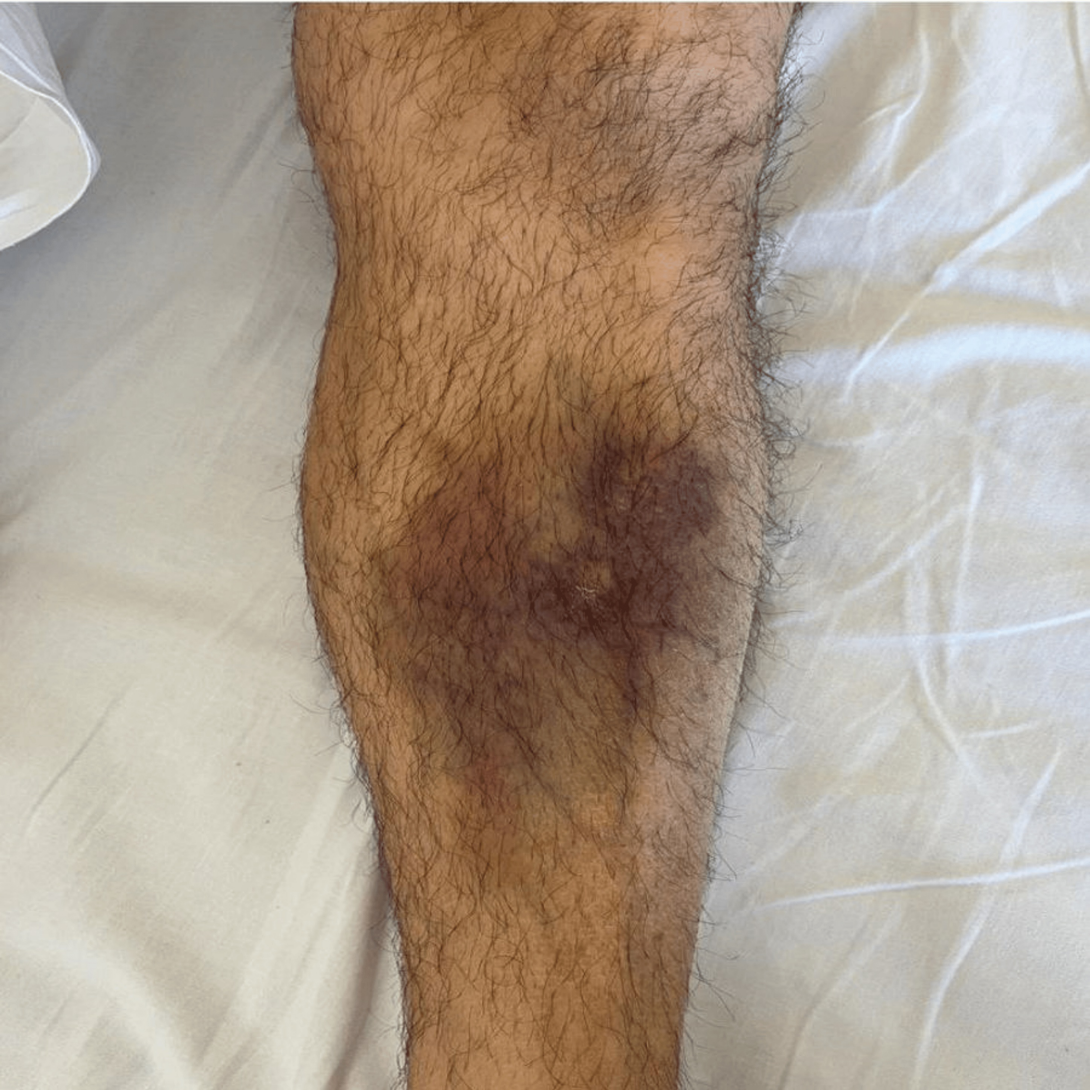
Ecchymosis on the leg of the patient

**Figure 2:**
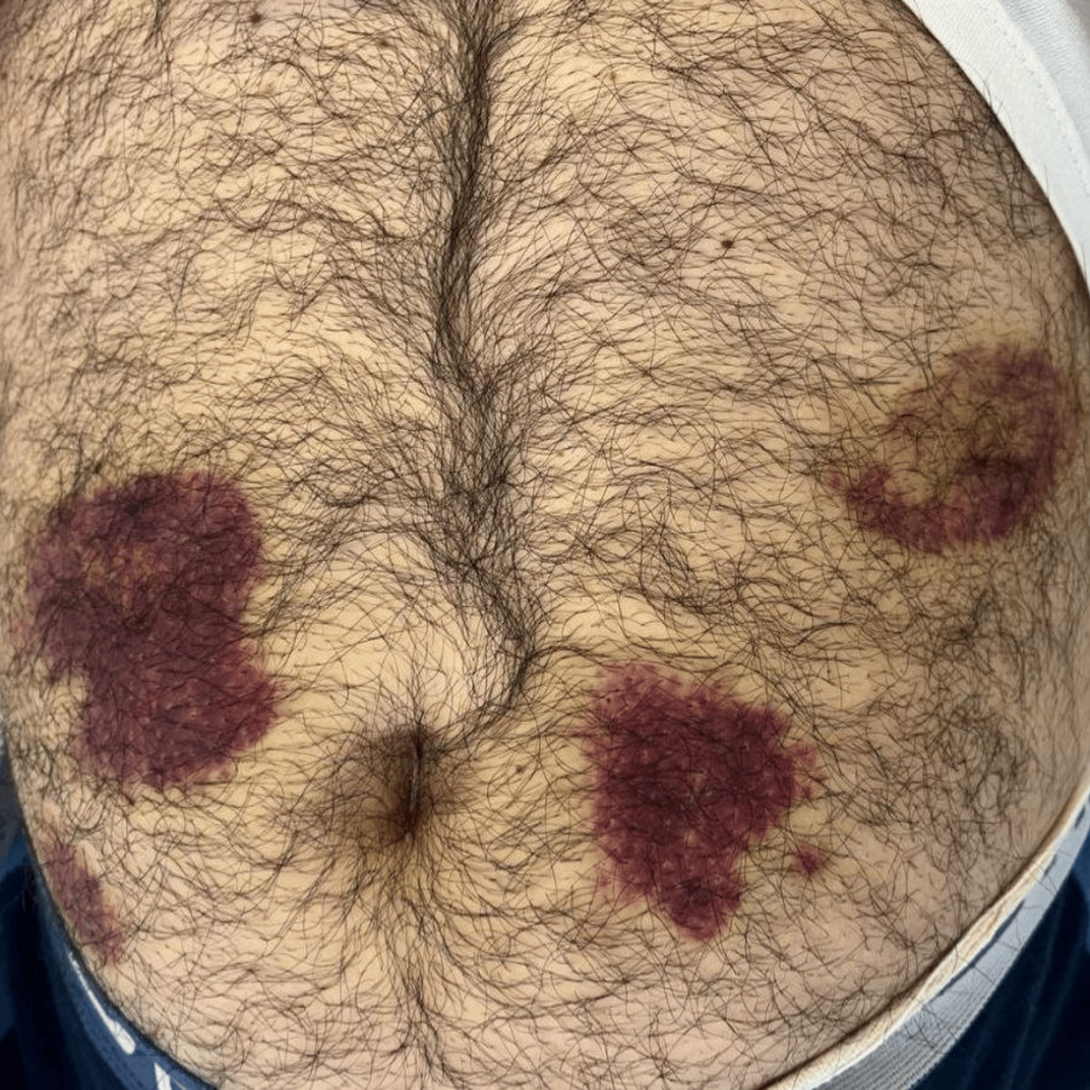
Ecchymosis on the abdomen of the patient

**Figure 3:**
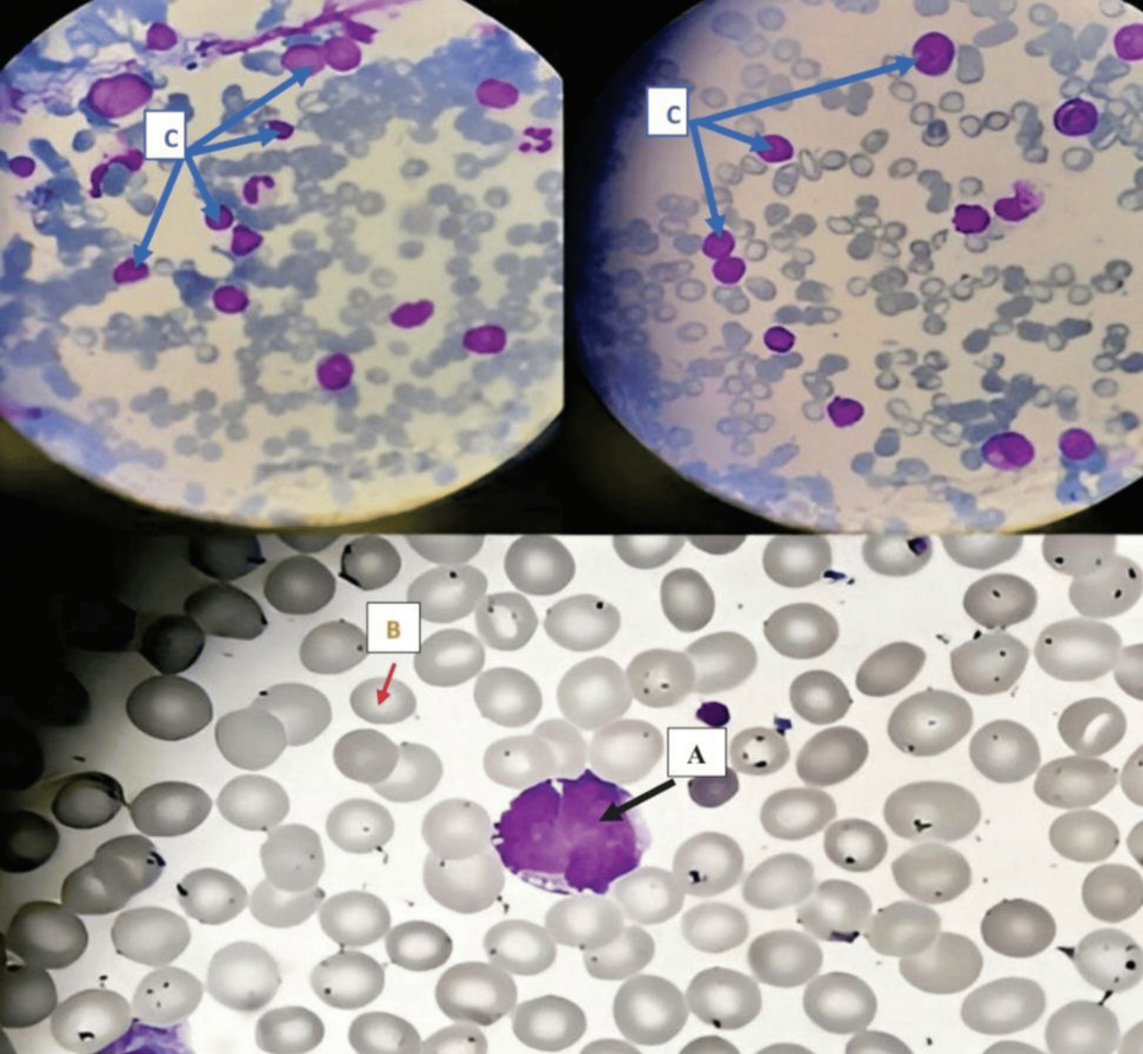
Bone marrow aspirate smears shows moderate cellularity with few spicules (small fragments around cells), suggesting probable hemodilution of the submitted sample. A (black arrow): There is predominance of blasts, including a majority of small-to-medium-sized cells with high nucleocytoplasmic ratio. B (red arrow): Nuclei are mostly rounded, some of them are clefted, and occasionally contain open chromatin whitish nuclei. No Auer rods are seen. Megakaryocytes are rare. C (Blue arrow): Erythropoiesis and granulopoiesis are represented by scattered elements. Plasma cells and lymphocytes are not increased in number.

In February 2024, the patient presented for evaluation at the emergency department due to back pain, and upon examination, a non-traumatic ecchymosis on the left leg and abdomen was revealed (Figures 1, 2). Initial laboratory assessments were significant for thrombocytopenia as well as elevated levels of D-dimer and lactate dehydrogenase (Table 1). Further workup via peripheral smear analysis identified atypical monocytoid cells, indicative of blast cells, comprising approximately 36% of the total cellular composition. Bone marrow aspiration (Figure 3) demonstrated moderate cellularity with blast predominance and small to medium-sized nuclei with high nucleocytoplasmic ratio, predominantly rounded, without Auer rods, consistent with acute leukemia. Immunophenotyping confirmed the diagnosis, showing positive expression for CD 10, CD 19, CD 22, CD 34, and HLA-DR markers, with partial expression of CD 20 and aberrant expression of CD 13. The cells tested negative for CD 38, further supporting the diagnosis of B-cell ALL. Additionally, genetic testing was positive for BCR-ABL t(9,22), further enhancing the diagnosis.

**Table 1:** Laboratory Investigations in our patient during hospitalization CD: Crohn's disease

Parameter	Patient Value	Reference range
Platelet count	100x10^9^	150-400x10^9^/L
D-Dimer	0.8 ug/ml	0.5 ug/ml
Lactate dehydrogenase	300	140-280 U/L
Peripheral blood blasts	36%	0%
Hemoglobin	10 g/dl	13.5-17.5 g/dl
White blood cell count	12x10^9^/L	4.0-11x10^9^/L
CD markers (immunophenotyping)	Positive for CD 10, 19, 20, 34, HLA-DR, aberrant CD 13	
BCR-ABL t(9,22)	Positive	Negative
Bone marrow blast percentage	Predominant	<5%

**Figure 4:**
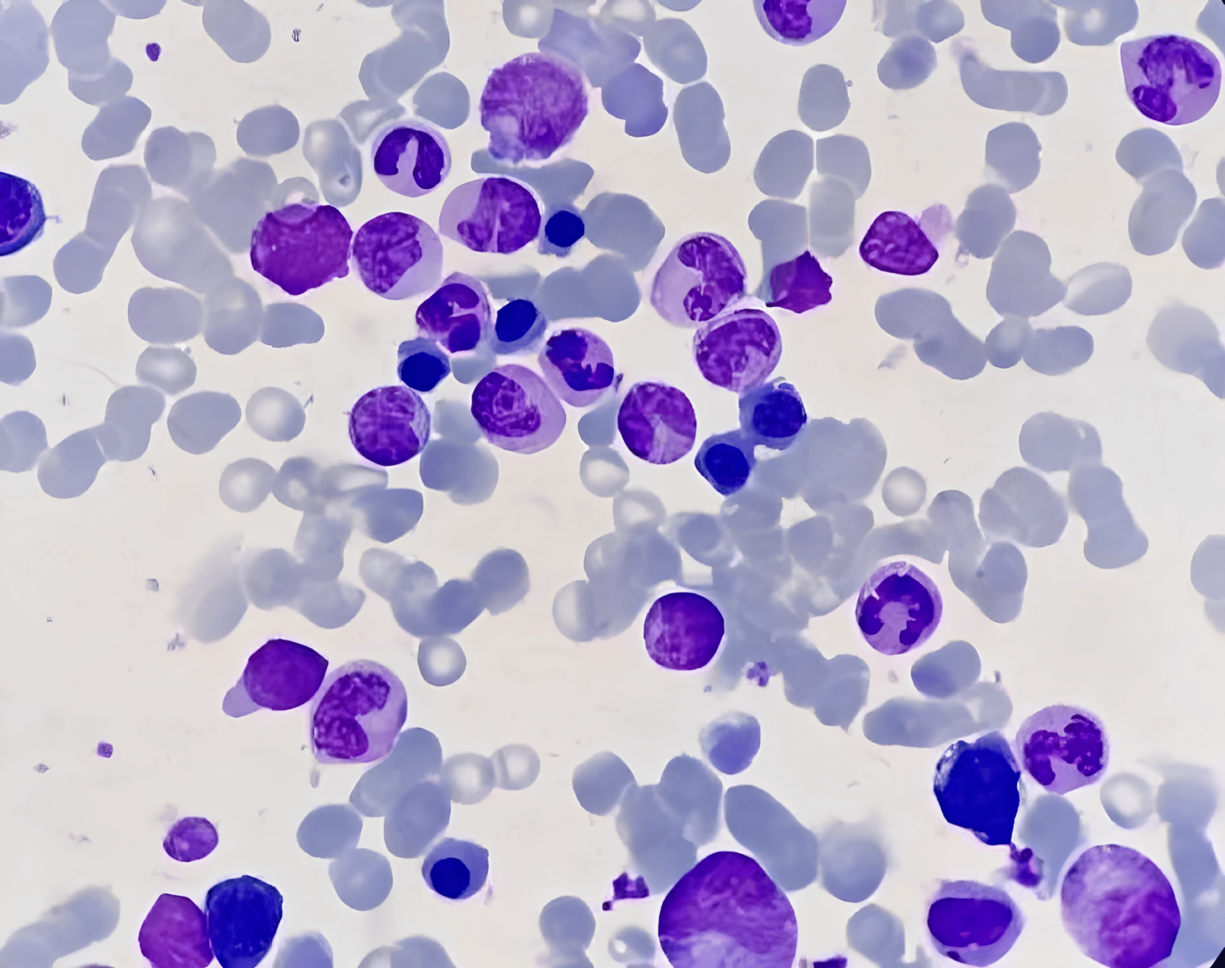
Normocellular bone marrow aspirate of our case of B-cell ALL, showing no increase in blast cells, most compatible with remission ALL: Acute lymphoblastic leukemia

Our patient received vedolizumab, anti-TNF therapy (infliximab), azathioprine, and a short period of prednisone during his illness, and was recently diagnosed with B-ALL with BCR-ABL(t9-22) positive. After undergoing two induction cycles of hyper-CVAD treatment, which comprises cyclophosphamide, vincristine sulfate, doxorubicin hydrochloride, and dexamethasone, the patient achieved remission. Bone marrow smears (Figure 4) obtained after induction revealed a normocellular bone marrow with no increase in blast cells, indicating remission. A decision was made to continue the consolidation treatment with the same regimen.

## Discussion

The emergence of therapy-related acute leukemia (t-AL) presents a significant concern not only among patients who had previously been treated with cytotoxic drugs for solid tumors or hematological malignancies but also among those with autoimmune diseases undergoing immunosuppressive therapy. While these treatments are pivotal for managing autoimmune conditions, they also carry an increased risk of secondary malignancies, particularly leukemia.

Autoimmune inflammatory diseases (AIDs), including CD and ulcerative colitis, are characterized by persistent, continuously activated inflammatory processes. This has been specifically linked to an elevated risk of developing myeloid malignancies [6]. Understanding the interplay between autoimmune diseases, immunosuppressive therapies, and the development of leukemia is essential to optimize patient care and prevent adverse outcomes.

The patient in this report developed t-ALL after 16 years of being on immunosuppressive treatment. Initially, he was treated with azathioprine for a period of six years, followed by infliximab, an anti-TNF agent, from 2014 to 2020 (six years). He then continued on vedolizumab for three years until it became unavailable, at which point it was substituted with prednisone until February 2024.

After a careful review, only a few cases exist in the literature of B-cell ALL patients who have previously undergone treatment with infliximab [7-11]. One such case, documented by Nergiz et al., involved a 22-year-old patient managing CD for three years. Subsequently, this patient was diagnosed with Ph (+) ALL following a period of azathioprine use, which lasted 10 months before being combined with infliximab for an additional 9 months. The total duration of infliximab treatment was 23 months [7].

Another case documented by Özdemirkiran et al. involved a 40-year-old patient managing ankylosing spondylitis for a decade. Subsequently, this patient was diagnosed with Ph (+) ALL following a nine-year course of treatment with indomethacin and sulfasalazine, as well as 10 months of each infliximab and adalimumab therapy [8].

In a separate case report by Cessani et al., a 62-year-old patient with a 23-year history of CD was diagnosed with Ph (+) ALL. The diagnosis followed 14 years of frequent steroid use alongside azathioprine and only four months of adalimumab treatment [9]. The case of the 40-year-old patient, as detailed by Alcain et al., revealed a relatively short, four-year history of CD. Due to gastrointestinal intolerance, this patient was unable to tolerate azathioprine treatment. Consequently, infliximab therapy was initiated after she showed no response to short-term methotrexate treatment. Shockingly, she was diagnosed with Ph (+) ALL after just 1.5 months of infliximab use [10].

It is important to highlight that the four cases of Ph (+) ALL had a prolonged history of chronic illness. One case represents a patient received prolonged anti-inflammatory treatment with salazopyrine, while the other three patients treated with long-term anti-TNF therapy, with one of them also undergoing immunomodulatory therapy with azathioprine.

A modest increase in the risk of MDS was found with any AID in a recent epidemiological investigation conducted by Wilson et al., showing a statistically significant odds ratio (OR) of 1.5 when compared to individuals without AID, particularly among those with AID for 10 years or longer, with an adjusted OR of 2.1 for MDS [12]. In a case-control investigation led by Nathalie et al., involving 40,011 individuals diagnosed with primary autoimmune diseases, 86 cases of confirmed therapy-related myeloid neoplasms were identified, comprising acute myeloid leukemia or myelodysplastic syndrome. The research aimed to assess the correlation between cytotoxic, anti-inflammatory, and immunomodulating medications and the likelihood of developing myeloid neoplasms in autoimmune disease patients. Remarkably, the study revealed a seven-fold increase in the risk of myeloid neoplasm associated with azathioprine exposure. Both control and case groups exhibited comparable systemic exposures across agent categories. However, no significant association was observed for anti-TNF agents. Additionally, no clear timeline was identified regarding the relationship between drug exposure and the incidence or development of myeloid neoplasm [13].

Anti-TNF and anti-α4β7 integrin therapies are nowadays increasingly used in CD.

In a systematic review of 28 studies (20 retrospective and 8 prospective), involving 298,717 IBD patients, infliximab was the most frequently used TNF inhibitors (TNFi). Both infliximab and adalimumab were evaluated in five studies.

In total, 692 malignancies were diagnosed in IBD patients treated with TNFi, accounting for an overall occurrence of 1.0%. The most frequent malignancies were non-melanoma skin cancers (123/692, 17.8%), digestive malignancies (120/692, 17.3%), and hematological malignancies (106/692, 15.3%).

The association between TNFi and malignancy was evaluated in 11 studies (39.3%); no significant association was found in ten studies, while an increased risk of lymphoma in patients exposed to TNFi was reported in one study [14]. Conversely, numerous cases of myeloid leukemia have been documented in patients with CD or other autoimmune conditions who were treated solely with anti-TNF drugs or had brief prior exposure to azathioprine. These instances may raise concerns about the potential of anti-TNF medications to exacerbate leukemia [15,16].

Additionally, anti-TNF therapy is frequently administered alongside azathioprine. Some suggestions have been made that this combination therapy carries a greater cancer risk compared to either anti-TNF or thioprine therapy alone [17].

However, malignancy data collected from both the GEMINI long-term safety (LTS) study and post-marketing surveillance indicate that the use of vedolizumab in the IBD population is not linked to an elevated incidence of malignancy [18].

## Conclusions

In conclusion, this case report describes Crohn's t-ALL with the BCR-ABL(t9-22) mutation in a patient from Lebanon. The involvement of immunosuppressive therapy and chronic immune-mediated inflammation may contribute to the development of leukemia. Further research is needed to evaluate risk factors for hematological malignancies in patients with IBD, particularly assessing the risks associated with anti-TNF therapy (infliximab) or vedolizumab when used alone or following treatment with thioprine.
